# Discordance of Species Trees with Their Most Likely Gene Trees

**DOI:** 10.1371/journal.pgen.0020068

**Published:** 2006-05-26

**Authors:** James H Degnan, Noah A Rosenberg

**Affiliations:** 1 Department of Biostatistics, Harvard School of Public Health, Boston, Massachusetts, United States of America; 2 Department of Human Genetics, Bioinformatics Program, and the Life Sciences Institute, University of Michigan, Ann Arbor, Michigan, United States of America; Harvard University, United States of America

## Abstract

Because of the stochastic way in which lineages sort during speciation, gene trees may differ in topology from each other and from species trees. Surprisingly, assuming that genetic lineages follow a coalescent model of within-species evolution, we find that for any species tree topology with five or more species, there exist branch lengths for which gene tree discordance is so common that the most likely gene tree topology to evolve along the branches of a species tree differs from the species phylogeny. This counterintuitive result implies that in combining data on multiple loci, the straightforward procedure of using the most frequently observed gene tree topology as an estimate of the species tree topology can be asymptotically guaranteed to produce an incorrect estimate. We conclude with suggestions that can aid in overcoming this new obstacle to accurate genomic inference of species phylogenies.

## Synopsis

Different genomic regions evolving along the branches of a tree of species relationships can have different evolutionary histories. Consequently, estimates of species trees from genetic data may be influenced by the particular choice of genomic regions used in an analysis. Recent work has focused on circumventing this problem by combining information from multiple regions to attempt to produce accurate species tree estimates.

The authors show that the use of multiple genomic regions for species tree inference is subject to a surprising new difficulty, the problem of “anomalous gene trees.” Not only can individual genes or genomic regions have genealogical histories that differ in shape, or topology, from a species tree, the gene tree topology *most likely* to evolve can differ from the species tree topology. As a result, the “democratic vote” procedure of using the most frequently observed gene tree topology as an estimate of the species tree topology can converge on the wrong species tree as more genes are added. As it becomes more feasible to simultaneously investigate many regions of a genome, species tree inference algorithms will need to begin taking the problem of anomalous gene trees into consideration.

## Introduction

In typical phylogenetic studies of individual genes, the estimated gene tree topology is used as the estimate of the species tree topology. When many loci are studied, the species tree topology is often estimated using the most frequently inferred gene tree topology [1–5]. Although it is well-known that the sorting of gene lineages at speciation can cause gene trees to differ in topology from species trees [6–9], the assumption that the most probable gene tree topology to be produced by this sorting is the same as the species tree topology—the implicit premise that makes it sensible to estimate a species tree using a single gene tree or the most common among several gene trees—has remained unquestioned. Here, under a population-genetic model for the evolution of gene lineages, we show that discordance can occur between the species tree and the most likely gene tree. Consequently, use of the most commonly observed gene tree topology to estimate the species tree topology—the “democratic vote” procedure among gene trees [10]—can be “positively misleading,” that is [11], convergent upon an erroneous estimate as the number of genes increases.

## Results

We refer to gene trees that are more likely than the tree that matches the species tree as *anomalous gene trees* (AGTs). To characterize the conditions under which AGTs exist, consider a rooted binary species tree *σ* with topology *ψ* and with a vector of positive branch lengths ***λ***, where *λ_i_* denotes the length of branch *i*. Following previous studies of gene trees and species trees [6,7,12–15], we use the coalescent process from population genetics [16,17] to model gene evolution in genetically variable populations along branches of a species tree. We consider gene trees that are known exactly, assuming that mutations have not obscured the underlying relationships among gene lineages.

For *n* species, and one gene lineage sampled per species, there are *n* − 2 internal branches of the species tree that affect gene tree probabilities under the coalescent. Branch lengths are measured in coalescent time units, which can be converted to units of generations under any of several choices for models of evolution within species [16–18]. In the simplest model for diploids, each species has constant population size *N*/*2* individuals, and *λ_i_* coalescent units equal *λ_i_N* generations.

We can view gene lineages as moving backward in time, eventually coalescing down to one lineage. In each interval, lineages entering the interval from a more recent time period have the opportunity to coalesce, with coalescence equiprobable for each pair of lineages—as specified by the Yule model [19–22]—and the coalescence rate following the coalescent process [16,17]. For the fixed species tree *σ*, the gene tree topology *G* is viewed as a random variable whose distribution depends on *σ*. Under the model, this distribution is known for arbitrary rooted binary species trees [15]. Using *P_σ_*(*G = g*) to denote the probability that a random gene tree has topology *g* when the species tree is σ, we define anomalous gene trees as follows.

### Definition 1

(i) A gene tree topology *g* is *anomalous* for a species tree σ = (*ψ, **λ***) if *P_σ_*(*G = g*) > *P_σ_*(*G = ψ*). (ii) A topology *ψ produces anomalies* if there exists a vector of branch lengths ***λ*** such that the species tree σ = (ψ, **λ**) has at least one anomalous gene tree. (iii) The *anomaly zone* for a topology *ψ* is the set of vectors of branch lengths ***λ*** for which *σ* = (*ψ, ***λ****) has at least one anomalous gene tree.

In other words, a gene tree topology *g* is anomalous for a species tree *σ* if a gene evolving along the branches of *σ* is more likely to have the topology *g* than it is to have the same topology as the species tree. AGTs do not exist for species trees with three taxa—the smallest number in a nontrivial, rooted, binary phylogeny. Denoting the length of the one internal branch in a three-taxon tree by *λ*, the probability is 1 − (2/3)*e^−λ^* that a gene tree has the same topology as the species tree [6,12,13]. This value always exceeds the probability that the gene tree topology matches one of the other two topologies, or (1/3)*e^−λ^.*

**Figure 1:**
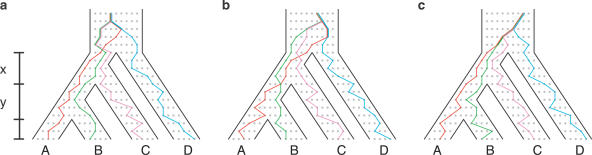
Anomalous Gene Trees for Four Taxa Colored lines represent gene lineages that trace back to a common ancestor along the branches of a species tree with topology (((AB)C)D). The figure illustrates how a gene tree can have a higher probability of having a symmetric topology, in this case ((AD)(BC)), than of having the topology that matches the species tree. If the internal branches of the species tree—*x* and *y*—are short so that coalescences occur deep in the tree, the two sequences of coalescences that produce a given symmetric gene tree topology together have higher probability than the single sequence that produces the topology that matches the species tree. (a) and (b) Two coalescence sequences leading to gene tree topology ((AD)(BC)). In (a), the lineages from B and C coalesce more recently than those from A and D, and in (b), the reverse is true. (c) The single sequence of coalescences leading to gene tree topology (((AB)C)D).

What about four taxa? If the species tree has sufficiently short branches, all coalescences of gene lineages may happen more anciently than its root. When coalescences are “deep,” the fact that random joining of lineages has a higher probability of producing some topologies than others [19,20,22] makes it likely that a gene tree has one of the high-probability topologies, regardless of the shape of the species tree. For four taxa, symmetric topologies each have probability 1/9, whereas asymmetric topologies each have probability 1/18 [6,19,20]. Thus, if the species tree is asymmetric with short branch lengths, symmetric gene tree topologies are more likely to be produced than are asymmetric topologies (Figure 1).

The set of branch lengths that lie in the four-taxon anomaly zone can be computed from the complete enumeration of probabilities for combinations of four-taxon gene trees and species trees [14,15]. For AGTs to occur with four taxa, the species tree must be asymmetric and the gene tree must be symmetric. To see that AGTs cannot occur with a symmetric four-taxon species tree, note that in Table 4 of Rosenberg [14], when the species tree has topology ((AB)(CD)), the terms for the probability that a gene tree has any four-taxon topology are subsumed among the terms for the probability of the topology ((AB)(CD)).

Suppose now that the species tree for the four taxa has the asymmetric topology (((AB)C)D). Let *x* be the length of the deeper internal branch and let *y* be the length of the shallower internal branch. Let *f*(*x,y*), *g*(*x,y*), and *h*(*x,y*) denote the probabilities for a gene tree evolving along this species tree to have topologies (((AB)C)D), ((AC)(BD)), and ((AB)(CD)), respectively. These functions can be obtained from Table 5 of Rosenberg [14], and they equal:

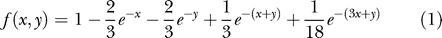


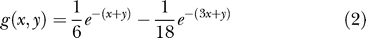


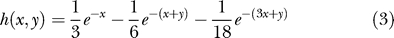
It is straightforward to show that for any positive values of *x* and *y*, *h*(*x,y*) > *g*(*x,y*). From this relationship, and from the fact that ((AC)(BD)) and ((AD)(BC)) are equiprobable gene tree topologies for a species tree with topology (((AB)C)D), it follows that the species tree gives rise to:

0 AGTs if *f*(*x,y*) ≥ *h*(*x,y*)
1 AGT if *g*(*x,y*) ≤ *f*(*x,y*) < *h*(*x,y*)
3 AGTs if *f*(*x,y*) < *g*(*x,y*).

Solving these inequalities, the species tree has

0 AGTs if *y* ≥ *a*(*x*)
1 AGT if *b*(*x*) ≤ *y* < *a*(*x*)
3 AGTs if *y* < *b*(*x*),

where the functions *a* and *b* are given as follows:


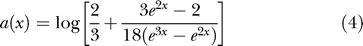



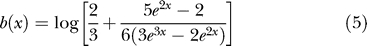


**Figure 2:**
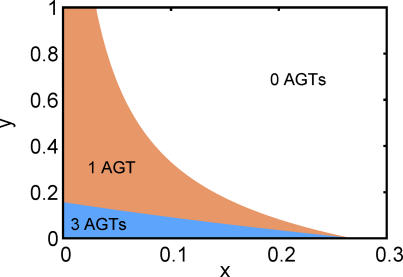
The Anomaly Zone for the Four-Taxon Asymmetric Species Tree Topology Branch lengths *x* and *y* (see Figure 1) are measured in coalescent time units.

Figure 2 illustrates the anomaly zone in the (*x,y*)-plane. For any *x*, at most one AGT occurs if *y* is greater than or equal to *b*(0) = log(7/6) ≈ 0.1542. For any *y*, no AGTs occur if *x* is greater than or equal to the solution to *a*(*x*) = 0, or approximately 0.2655. For small *x*, AGTs are produced even for large *y*; as *x* approaches 0, *a*(*x*) approaches ∞, showing that very short branches deep in the species tree can lead to AGTs even if recent branches are long.

What happens with more than four taxa? Although for four taxa, symmetric topologies do not produce anomalies, for five or more taxa, *every* species tree topology—including those that are highly symmetric—produces anomalies. In other words, for any species tree topology with *n* ≥ 5 taxa, there is a region of the space of branch lengths in which the gene tree topology most likely to occur differs from the species tree topology. We state this result as Proposition 2, and we use Definition 3 and Lemmas 4 and 5 for the proof.

### Proposition 2

Any species tree topology with *n* ≥ 5 taxa produces anomalies.

### Definition 3

A labeled topology *L_n_* for *n* taxa is *n-maximally probable* if its probability under the Yule model of random branching [19–22] is greater than or equal to that of any other labeled topology for *n* taxa.

### Lemma 4

For any *n* ≥ 4, any species tree topology that is not *n*-maximally probable produces anomalies.

### Lemma 5

For any *n* ∈ {5,6,7,8}, any species tree topology that is *n*-maximally probable produces anomalies.

### Overview of Proofs

We will return to the proofs of the lemmas. For Lemma 4, the idea is that species tree branch lengths can be made short enough that with high probability, all coalescences of gene lineages occur more anciently than the species tree root; the gene tree is then more likely to have a maximally probable labeled topology than to have the topology of the species tree. Lemma 5 is proven by finding the *n*-maximally probable topologies for *n* ∈ {5,6,7,8}, and by showing that each of them produces an anomaly.

Assuming the lemmas, what must be shown is that for any *n* ≥ 9, any *n*-maximally probable species tree topology produces anomalies. The idea of the proof is to use the strong induction principle. Any *n*-maximally probable species tree topology consists of two subtrees immediately descended from the root. By the inductive hypothesis, the labeled topology for one of these subtrees produces anomalies. Branch lengths can then be chosen for the tree of *n* species so that the gene lineages in one of the subtrees are likely to give rise to an AGT, and so that the lineages in the other subtree are likely not to do so. With these branch lengths, the species tree topology has an AGT.

### Proof of Proposition 2

By Lemmas 4 and 5, for 5 ≤ *n* ≤ 8, any species tree topology with *n* taxa produces anomalies. Given *N* ≥ 8, suppose that for 5 ≤ *n* ≤ *N*, anomalies are produced by any species tree topology with *n* taxa. It must be shown that this implies that any species tree topology with *N* + 1 taxa produces anomalies. For species tree topologies that are not (*N* + 1)-maximally probable, this is accomplished using Lemma 4.

Consider an (*N* + 1)-maximally probable species tree topology *ψ*, where *N* ≥ 8. To show that *ψ* produces anomalies, we construct branch lengths for a species tree *σ* with labeled topology *ψ*. For one of the two subtrees immediately descended from the root of *σ*, the number of taxa in the subtree must be in *W* = {5,6,…,*N*}. Denote this subtree by *S*, and the other subtree immediately descended from the root by *S′* (if the numbers of taxa in the two subtrees are both contained in *W*, then the choice for *S* is arbitrary). These subtrees have labeled topologies *L_S_* and *L_S′_*, respectively.

By the inductive hypothesis, the labeled topology *L_S_* of *S* produces anomalies. That is, there exists a set of branch lengths ***B*** *_S_* and a labeled topology L^*^ such that if a species tree has topology *L_S_* and branch lengths ***B*** *_S_*, the probability of a gene tree having labeled topology L^*^, or *q_2_*, is greater than that of the gene tree having labeled topology *L_S_*, or *q_1_*. This assumes that the gene lineages from *S* are the only lineages present to coalesce.

Choose the internal branch lengths ***B*** *_S′_* of *S′* and the length *B′* of the branch connecting the root of *S′* and the root of *σ* to be long enough that the probability that each coalescence in the gene tree occurs along the first branch of the species tree where it is possible to occur exceeds 1 − *α*, where *α* < 1 − *q_1_/q_2_*. In other words, the probability that the gene tree for *S′* (with branch lengths ***B*** *_S′_*) has labeled topology *L_S′_* and most recent common ancestor (MRCA) more recent than the root of *σ* is at least 1 − *α*.

Let *ɛ* < [(1 − *α*)*q_2_* − *q_1_*]/(2 − *α*). Choose the branch lengths of subtree *S* to correspond to ***B*** *_S_*, and let the length *B* of the branch connecting the root of *S* and the root of *σ* be sufficiently long that the probability that all gene lineages from *S* coalesce more recently than the root of *σ* exceeds 1 − *ɛ*. Increase *B′* or *B* as needed so that the root of *σ* is located where these branches intersect.

The probability that a gene tree on *σ* matches the species tree in labeled topology is at most (1)[(*ɛ*)(1) + (1)(*q_1_*)], where the terms arise as follows: (1) is an upper bound on the probability that all coalescences from *S′* occur more recently than the root of *σ* and are compatible with the species tree topology; (*ɛ*)(1) is an upper bound on the probability that at least one coalescence from *S* occurs more anciently than the root of *σ* (*ɛ*), times the maximal probability of the gene tree topology matching *ψ* in this setting (1); and (1)(*q_1_*) is an upper bound on the probability that all coalescences from *S* occur more recently than the root of *σ* (1), times an upper bound on the probability of the gene tree topology matching *ψ* in this setting (*q_1_*). This probability has *q_1_* as an upper bound because the probability of the gene tree topology matching *ψ* is less than or equal to the probability that the gene tree topology for the lineages in *S* matches *L_S_*.

The probability that a gene tree for *σ* has a labeled topology ψ^*^ whose two subtrees immediately descended from the root have labeled topologies *L_S′_* and L^*^ is at least (1 − *α*)(*q_2_* − *ɛ*). Here 1 − *α* is a lower bound on the probability that all coalescences from *S′* occur more recently than the root of *σ* in a manner compatible with the species tree topology, and *q_2_* − *ɛ* is a lower bound on the probability that all coalescences from *S* occur both more recently than the root of *σ* and in a manner compatible with topology L^*^. This lower bound equals *q_2_* − *ɛ* as the difference between the probability that the lineages from *S* would have labeled topology L^*^ if allowed to proceed to coalescence without other lineages being present (*q_2_*) and the upper bound on the probability that other lineages become available for coalescence, that is, the upper bound on the probability that coalescence happens more anciently than the root of *σ* (*ɛ*).

The choice of *ɛ* guarantees that (1 − *α*)(*q_2_* − *ɛ*) > *ɛ* + *q_1_*. Thus, for species tree *σ*, gene tree topology ψ^*^ is more probable than *ψ*, and *ψ* therefore produces anomalies.

### Proof of Lemma 4

Consider a species tree that has *n* species and a labeled topology *L* that is not *n*-maximally probable. The probability that no coalescences of gene lineages in a gene tree on the species tree occur more recently than the species tree root can be bounded below as follows. The species tree has *n* − 2 internal branches, where the length of branch *i* is *λ_i_* coalescent time units. If *n_i_* is the number of lineages “entering” branch *i* (that is, the number available for coalescence on branch *i*), the probability that the *n_i_* lineages coalesce to *j* lineages during coalescent time *λ_i_* is a known function *p_n_i___,j_*(λ*_i_*) [17,23,24], among whose properties are lim_λ_i___→∞_ *p*_*n*_*i*___,1_(λ*_i_*) = 1 and lim_*λ_i_*__→0_ *p_n_i_,n_i__*(λ*_i_*) = 1.

Because 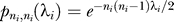 , 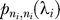 decreases as *n_i_* and *λ_i_* increase. Therefore, denoting 
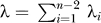
 , the probability of no coalescences on any internal branch is


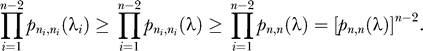


Let *q_1_* be the probability under the Yule model that a gene tree has labeled topology *L*, and let *q_2_* be the probability that a gene tree has the *n*-maximally probable labeled topology *M*. Because *L* is not *n*-maximally probable, *q_2_* > *q_1_*. For *ɛ* > 0, because lim_*λ*→0_ *p_n,n_*(λ) = 1, *λ* can be chosen small enough that *p_n,n_*(λ) > (1 − *ɛ*)^1/(*n* − 2)^, so that the probability that no coalescences occur on any internal branch (and all coalescences occur more anciently than the root) is greater than 1 − *ɛ*.

Let *ɛ* < (*q_2_* − *q_1_*)/(*q_2_* + 1). The probability that a gene tree on the species tree has labeled topology *L* is less than *ɛ* + *q_1_*, as the probability that at least one coalescence occurs more recently than the root of the species tree is less than *ɛ*, and if all coalescences occur more anciently than the root, the probability is *q_1_* that the gene tree has labeled topology *L*.

The probability that a gene tree on the species tree has labeled topology *M* is greater than (1 − *ɛ*)*q_2_*, as the probability that all coalescences occur more anciently than the species tree root is greater than 1 − *ɛ*, and if all coalescences occur more anciently than the root, the probability is *q_2_* that the gene tree has labeled topology *M*. The choice of *ɛ* guarantees that (1 − *ɛ*)*q_2_* > *ɛ* + *q_1_*, from which it follows that topology *L* produces anomalies.

### Proof of Lemma 5

To identify the *n*-maximally probable labeled topologies for *n* ∈ {5,6,7,8}, the probability of each labeled topology *L* can be calculated as 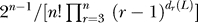 , where *d_r_*(*L*) is the number of internal nodes in the topology that have exactly *r* descendants (Table 1) [20,22]. It now must be shown that each of these *n*-maximally probable topologies produces anomalies.

**Table 1:**
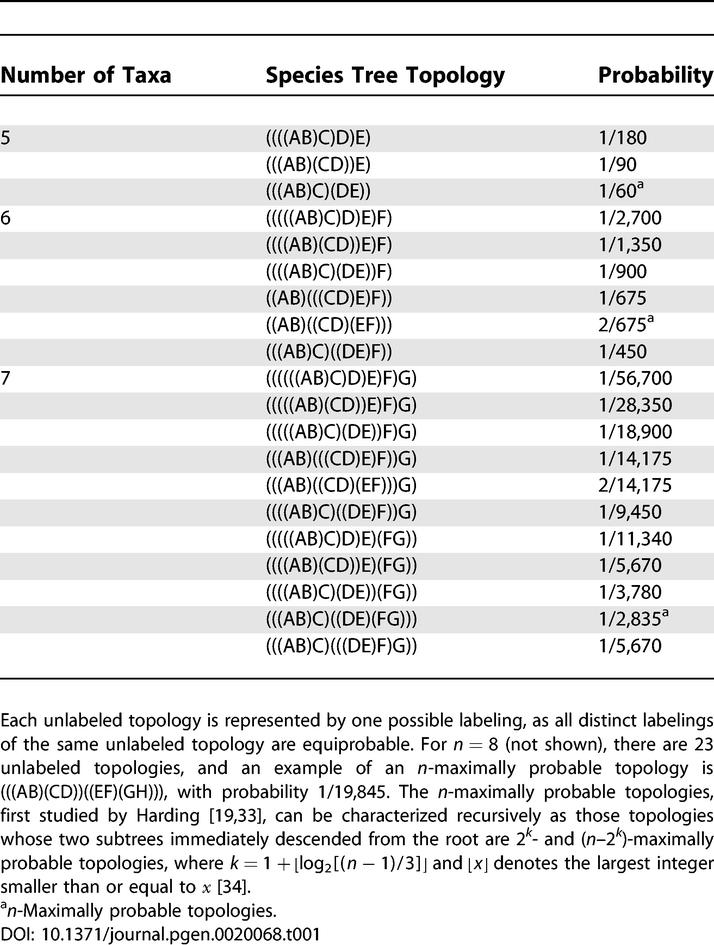
*n*-Maximally Probable Topologies for *n* = 5, 6, 7

**Figure 3:**
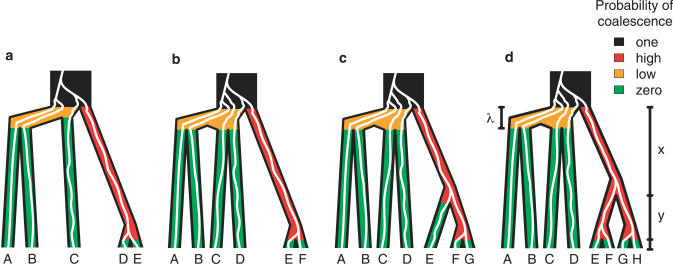
The Production of Anomalies for *n*-Maximally Probable Species Tree Topologies with *n* = 5,6,7,8 (See Table 1) The branch lengths *x*, *y*, and *λ* apply to each tree: in (a) and (b), *x* + *y* denotes the length of the red internal branch, and in (c) and (d), *x* and *y* are the lengths of the deeper and shallower red internal branches, respectively; the length *λ* denotes the branch length between the root of the species tree and the MRCA of species A and B. For each tree, the color of a branch represents the probability that coalescences occur on the branch. On an external branch, because there is only one gene lineage, coalescences cannot occur. Prior to the root, the probability is 1 that all lineages coalesce. During the time between the root of the species tree and the divergence of A and B—and of C and D in (b–d)—the probability that any coalescences occur can be made arbitrarily close to 0 by making the internal branches sufficiently short. Similarly, by choosing *x* and *y* to be sufficiently large, the probability that all available lineages coalesce on the red branches can be made arbitrarily close to 1. In (a), the species tree can be represented as (((AB)C)Z), where Z is (DE). By making the internal branch ancestral to D and E long, the subtree Z is similar to a single taxon, and the five-taxon tree behaves like the four-taxon asymmetric tree (((AB)C)Z), which produces the anomaly ((AB)(CZ)). Thus, in (a), the AGT is ((AB)(C(DE))). Similarly, the species tree topologies in (b), (c), and (d) have the form (((AB)(CD))Z) and produce anomalies (((AB)C)(DZ)); in (b), (c), and (d) Z is (EF), (E(FG)), and ((EF)(GH)), respectively. The anomalies occur by letting internal branches in subtrees ((AB)(CD)) and Z be sufficiently short and long, respectively.

Consider the species trees in Figure 3. Let *x* and *y* denote lengths of internal branches, as shown in the figure. For each tree, let *λ* be the total time between the root and the MRCA of A and B. (For *n* = 6,7,8, we can assume without loss of generality that the MRCA of C and D is at least as ancient as the MRCA of A and B.) For *n* = 5 and *ɛ* > 0, *λ* can be made short enough and *x* + *y* large enough so that when the species tree root is reached, the probability is at least 1 − *ɛ* that the gene lineages from species D and E have coalesced and that no other coalescences have occurred. The probability that the gene tree matches the species tree is at most *ɛ* + (1 − *ɛ*)(1/18), and the probability that its topology is ((AB)(C(DE))) is at least (1 − *ɛ*)(1/19). For *ɛ* < 1/19, the species tree topology produces an anomaly.

For *n* = 6 and *ɛ* > 0, *λ* can be made small enough and *x* + *y* large enough that when the species tree root is reached, the probability is at least 1 − *ɛ* that the gene lineages from species E and F have coalesced and that no other coalescences have occurred. The probability that the gene tree matches the species tree is at most *ɛ* + (1 − *ɛ*)(1/90), and the probability that its topology is (((AB)C)(D(EF))) is at least (1 − *ɛ*)(1/60). For *ɛ* <1/181, the species tree topology produces an anomaly. For *n* = 7 and *n* = 8, the proof follows the same argument as for *n* = 6 but with *x* and *y* both large, and with AGTs of (((AB)C)(D(E(FG)))) and (((AB)C)(D((EF)(GH)))), respectively.

## Discussion

We have shown that all species tree topologies with five or more taxa, as well as asymmetric topologies with four taxa, have anomaly zones, regions in branch length space in which the most frequently produced gene tree differs from the species tree topology. In this region, assuming that gene trees are known exactly, the “democratic vote” procedure of using the most common gene tree as the estimate of the species tree is statistically inconsistent for phylogenetic inference. This inconsistency has a noticeable parallel with the inconsistency of maximum parsimony methods for inferring gene trees [11], as both settings experience a transition when the number of taxa *n* reaches five. Under the assumption of equal evolutionary rates throughout a tree, only if *n* ≥ 5 can parsimony be inconsistent [25], and under the model we have studied for gene tree evolution along the branches of species trees, AGTs—although they can occur for *n* = 4 with asymmetric species tree topologies—occur for *all* species tree topologies only if *n* ≥ 5.

Species trees with at least one short branch, especially if it is deep in the tree, are particularly susceptible to producing AGTs. For an asymmetric species tree with four taxa, by solving *a*(*x*) ≤ *x*, it can be seen that the anomaly zone includes the region in which both internal branch lengths are below ≈0.156 coalescent time units, or 0.156*N* generations if the species along these branches were constant-sized diploid populations with effective size *N*/2 individuals. However, if the deeper internal branch is shorter than 0.156 coalescent units, the shallower internal branch can become much longer without exiting the anomaly zone.

Anomalous gene trees might not exist for typical four-taxon species trees, as branch lengths of 0.1–0.2 coalescent units are probably small compared to the time scale of most speciations. For example, for the human-chimp-gorilla-orangutan tree, Rannala and Yang [26] obtained an estimate of 1.2 million y for the shorter of the two internal branches in the tree, namely the branch separating the divergence time of humans and chimpanzees and the more ancient divergence of gorillas from the human-chimp lineage. Using their estimate of 24,600 for the effective size *N*/2 and 20 y for the generation time, this value translates into 1.2 coalescent units. Although there is considerable uncertainty in each aspect of the calculation, it seems unlikely that AGTs arise for the human-chimp-gorilla-orangutan tree.

If the number of taxa considered is large, however, the AGT problem may be quite severe, as species trees with many taxa typically contain some deep short branches. This is especially true as taxonomic sampling increases, because the addition of taxa within a monophyletic group necessarily shortens some internal branches. Thus, AGTs are more likely to complicate inference for such speciose and rapidly diverging groups as *Drosophila,* in which large effective population sizes may have caused intervals between speciations to be relatively short in coalescent time. AGTs may also result from adaptive radiations, during which many divergences may have occurred in rapid succession, and from population divergences in population genetics and phylogeography, as population trees generally involve very closely related groups.

**Figure 4:**
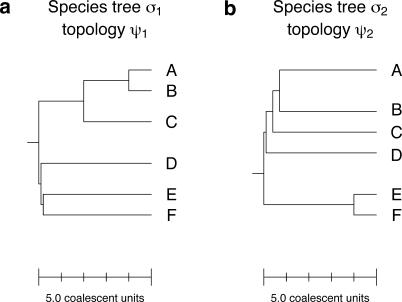
A Wicked Forest (a) The two long internal branches have length 2, and the two short internal branches have length 0.1. For this species tree the probabilities that a random gene tree has topology *ψ*_i_ are 0.085 and 0.103 for *i* = 1 and *i* = 2, respectively. Hence *ψ*_2_ is anomalous for *σ*_1_. (b) The one long internal branch has length 4, the shortest internal branch has length 0.1, and the other two internal branches have length 0.3. For this species tree, the gene tree probabilities are 0.066 and 0.060 for topologies *ψ*_1_ and *ψ*_2_, respectively. Note that the two topologies disagree only on the placement of taxon D and that neither is 6-maximally probable.

Although AGTs are easiest to find when the gene tree has more symmetry than the species tree, a consequence of Proposition 2 is that an AGT can have *less* symmetry than its underlying species tree. Additionally, a set (or *forest*) *W* of species trees can exhibit a surprising form of mutual anomalousness (Figure 4). We refer to a set *W* of at least two trees as a *wicked forest* if *σ_i_, σ_j_* ∈ *W* and *i* ≠ *j* imply that the topology of *σ_i_* is anomalous for *σ_j_*. By choosing one of the trees in a set to be *n*-maximally probable, it is not difficult to find examples of wicked forests, and although the example in Figure 4 has two trees, wicked forests can also be found that contain three or more trees (not shown). The counterintuitive result is that if two trees from the same wicked forest were considered as hypotheses for a phylogeny, observing a higher proportion of gene trees that match one species tree would be evidence in favor of the other species tree, and vice versa.

It is noteworthy that our theoretical results apply to known—rather than estimated—gene trees, and do not consider the effect of mutations on inference of gene trees. This issue is important, as mutational history is a key factor in determining when an empirical study might actually be misled by AGTs. As an illustration, in one human-chimp-gorilla study, a substantial fraction of loci—six of 45 considered—had no informative substitutions that could provide support to any particular phylogenetic grouping [3]. That this many loci would not have any phylogenetic information in the human-chimp-gorilla clade suggests that for the smaller branch lengths typical of the anomaly zone, the fraction of uninformative loci could be much greater. Thus, situations that give rise to AGTs may coincide largely with situations for which the history of mutation does not produce enough informative sites to allow multifurcations in estimated gene trees to be resolved into sequences of bifurcations. However, the occurrence of informative sites depends on other factors besides those that lead to AGTs, such as external branch lengths of the species tree and rates of mutation and substitution; consequently, high substitution rates for species trees in the anomaly zone may very well lead to production of detectable AGTs. Just as an average over species trees generated from a speciation model can be used to assess how often maximum parsimony is inconsistent [27], such an analysis could be used to evaluate the frequency with which realistic species trees give rise to AGTs. Of particular interest will be the extent to which AGTs occur at branch lengths and substitution rates for which the effects of mutation do not render gene trees unrecoverable; for species trees with these parameter values, empirical phylogenetic studies could be misled specifically by AGTs rather than by other difficulties in estimation.

What implications do AGTs have for the design of phylogenetic studies? First, their existence demonstrates that adding more genes to a phylogenetic analysis will not necessarily improve the inference, unless this approach is combined with algorithms that avoid the problem of AGTs. The commonly used concatenation procedure [28,29]—in which the species tree is inferred by concatenating a set of loci and then employing the resulting sequence alignment to estimate a single gene tree—is not immune to the AGT problem (L. S. Kubatko and J. H. Degnan, unpublished data). Other types of data, such as inversions or genomic rearrangements, also would not necessarily help, as our results apply to any traits that evolve genealogically.

One strategy that may circumvent the occurrence of AGTs is the use of a sample with multiple individuals per species. Because many lineages from each species may persist reasonably far into the past, the chance of coalescences on a short branch is higher if many lineages are present [7,14,30]. Thus, increasing the sample size has a similar effect to lengthening short branches near the tips. As multiple sampled lineages from a species will coalesce on recent branches of the species tree, however, increased sample sizes will not assist the inference if recent branches are long but deep branches in the species tree are short.

Additionally, because AGTs are absent for sets of three species, a sensible approach may be to use many genes to decisively infer all *_n_C*_3_ species trees for sets of three species, and to then use the uniqueness of species trees given their three-taxon clades [31,32] for species tree inference. Different algorithms for combining data on multiple loci will have different degrees of susceptibility to the occurrence of AGTs, and a challenge for phylogenetics is to identify those procedures that are best able to overcome this new obstacle to accurate inference of species trees.

## Materials and Methods

The methods used are included in the Results section.

## Abbreviations

AGT: anomalous gene tree
MRCA: most recent common ancestor
